# Long-Term Effects of a Short Juvenile Feeding Period with Diets Enriched with the Microalgae *Nannochloropsis gaditana* on the Subsequent Body and Muscle Growth of Gilthead Seabream, *Sparus aurata* L.

**DOI:** 10.3390/ani13030482

**Published:** 2023-01-30

**Authors:** María Dolores Ayala, Noemí Balsalobre, Elena Chaves-Pozo, María Isabel Sáez, Alba Galafat, Francisco Javier Alarcón, Tomás Francisco Martínez, Marta Arizcun

**Affiliations:** 1 Faculty of Veterinary, Department of Anatomy and Comparative Pathological Anatomy, Campus of Espinardo, University of Murcia, 30100 Murcia, Spain; 2 Instituto Español de Oceanografía, Centro Oceanográfico de Murcia (COMU-IEO), CSIC, Puerto de Mazarrón, 30860 Murcia, Spain; 3 Departamento de Biología y Geología, Universidad de Almería, CEIMAR, 04120 Almería, Spain

**Keywords:** long term growth, microalgae diets, muscle cellularity, *Sparus aurata*

## Abstract

**Simple Summary:**

Currently, microalgae are used as a partial substitute for fishmeal and fish oil in fish diets, but it is unknown if this can influence the long-term growth of the fish. In the present work, we have studied the long-term effects of diets enriched with the microalgae *Nannochloropsis gaditana* that were supplied to juvenile gilthead seabream, *Sparus aurata*, at two inclusion levels (2.5 and 5%), either raw (R2.5 and R5 groups) or cellulose-hydrolyzed (H2.5 and H5 groups), for three months. Subsequently, all groups were transferred to a microalgae-free diet until commercial size was reached (1.6-year-old, ≈27 cm and ≈300 g). The results showed that three-month microalgae supplementation of the diets in juvenile fish had a long-term influence on the final body and muscle growth, as well as in the fibrillar constitution of the myotome. This influence depended on the state (raw versus hydrolyzed) and the microalgae inclusion levels in the diet, in such a way that the hydrolyzed diet mainly increased muscle growth and hyperplasia of the specimens, and this was greater at higher inclusion levels. The fact that microalgae inclusion in the juvenile diet can improve subsequent growth may be advantageous for fish farmers.

**Abstract:**

Currently, microalgae are used in fish diets, but their long-term growth effect is unknown. In this experiment, juvenile seabream specimens were fed with microalgae-enriched diets for three months, and then transferred to a microalgae-free diet for 10 months to assess long-term effects up to commercial size (≈27 cm and ≈300 g). The juvenile diets contained *Nannochloropsis gaditana* at 2.5 or 5% inclusion levels, either raw (R2.5 and R5 groups) or cellulose-hydrolyzed (H2.5 and H5 groups). The body length and weight were measured in 75 fish group^−1^ at commercial stage. The size, number, and fibrillar density of white muscle fibers and the white muscle transverse area were measured in nine fish group^−1^ at commercial stage. The results showed the highest body weight in H5 at commercial stage. The white muscle transverse area and the white fibres hyperplasia and density also showed the highest values in H5, followed by H2.5. In contrast, the highest hypertrophy was observed in C and R2.5, being associated with the lowest muscle growth in both groups. These results showed a microalgae concentration-dependent effect in hydrolyzed diets as well as an advantageous effect of the hydrolyzed versus raw diets on the long-term growth of *Sparus aurata*.

## 1. Introduction

Gilthead seabream, *Sparus aurata* L., is a carnivorous teleost fish widely distributed in the Mediterranean and Atlantic seas. This species is a fast-growing commercial fish of great value for Mediterranean aquaculture. Currently, alternative protein and lipid sources to fishmeal and fish oil are being used in aquaculture due to the inconveniences that they entail: unsustainability, rising prices, and the need of obtaining them from extractive fishing, lipid oxidation of their fatty acids, etc. [1,2]. For this reason, fishmeal and fish oils are currently being partially replaced by products of plant origin. Removing fishmeal from the diets of omnivorous species has been readily achieved, but this has been more difficult to implement in carnivorous fish and crustaceans [3]. It has been generally found that up to 50% fishmeal protein can be replaced by plant proteins in carnivorous fish diets without any negative effects on growth or fish welfare issues [2]. However, when aquafeeds have a percentage of vegetable protein greater than 50%, some studies have observed that the fish growth of some species is inferior to that of fish fed fishmeal-based diets [4,5]. This is due to the presence of anti-nutritional factors in vegetable proteins, indigestible carbohydrates, as well as less efficiency in protein digestion and amino acid absorption. In addition, the plant oil sources used are poor in long-chain polyunsaturated fatty acids (PUFAs) [6]. All these factors entail disadvantages when an excess of terrestrial plants is used in the diet and can compromise fish health and nutritional value of fillets for human consumption [7,8,9,10]. 

Currently, microalgae represent a promising candidate to partially replace terrestrial vegetables in aquafeeds. Overall, protein content of microalgae ranges from 30 to 55% (on a dry matter basis) [11]. Furthermore, microalgal protein shows comparable amino acid profiles among different species, characterized by a high content in essential amino acids, as reported by the comprehensive study of 40 species of microalgae by Brown et al. [12]. The lipid content of microalgae ranges from 2% to 50% on a dry matter basis and they are rich in polyunsaturated fatty acids [13]. With the improvement of fatty acids profile, the aquatic animals will have higher nutritional values and positively impact the consumers’ health. Further, supplementation of microalgal PUFAs in diet could promote the growth and enhance the immune response of aquatic animals [14,15]. In addition, microalgae are rich in bioactive compounds, such as pigments, polyphenols, and vitamins, among others [12,16,17]. Different studies have pointed out that these bioactive substances can exert positive effects on several aspects of fish physiology, even if added at a low inclusion level (e.g., less than 10%) in feeds [18,19]. Given their current production costs, the interest in microalgae is turning from large-scale use as a main ingredient towards their use as functional additives at low inclusion levels in feeds [20].

Among the main microalgae species that are cultivated as aquaculture feed, we can highlight: *Isochrysis, Nannochloropsis, Pavlova, Phaeodactylum, Chaetoceros, Thalassiosira, Tetraselmis, and Rhodomonas*. The species that are used in aquaculture are selected following criteria such as the absence of toxic compounds, their nutritional values, and good digestibility for the species to be fed. In this context, *Nannochloropsis gaditana*, by virtue of its richness in eicosapentanoic acid (EPA, C20:5n-3), pigments, and other natural antioxidants and bioactive compounds [21,22,23], together with its availability at the industrial or semi-industrial scale [24,25], is a promising candidate as a commercial additive in Aquafeed [20]. So far, numerous studies have reported positive effects of microalgae-enriched diets on fish growth [26,27,28,29,30,31,32]. However, studies on the influence of microalgae on fish muscle growth are still very scarce [33]. The skeletal muscle is the main constituent of the edible part of fish commercial species. Skeletal muscle structure strongly influences the organoleptic properties of the fillet, such as colour and—especially—texture [34,35,36,37], and it can be influenced by both extrinsic and intrinsic factors [37]. One of the most widely recognised exogenous factors is the diet [38,39]. Hence, those factors able to modify the muscle structure can have a considerable impact on fillet quality. Fish skeletal muscle growth occurs through a double mechanism that involves the formation of new fibres (hyperplasia) and the increase in volume of pre-existent fibres (hypertrophy). Whereas hypertrophy is based on increased protein synthesis and on the incorporation of nuclei to keep the nuclei/cytoplasm ratio, hyperplasia requires the capacity of muscle fibre formation to go beyond embryonic development [40]. Ayala et al. [41] studied the effects of dietary supplementation with the microalgae *Nannochloropsis gaditana* for three months on juvenile sea bream growth. To that end, a control microalgae-free diet (C) was compared with four experimental diets, in which the protein content of plant origin was partially replaced by raw biomass of the microalga *N. gaditana* at 2.5 and 5% inclusion levels, or by enzymatically prehydrolyzed *N. gaditana* at 2.5 and 5%. Microalgal biomass enzyme pre-treatment with cellulase was performed to increase the bioavailability of the cellular inner components. The results showed that microalgae not only lacked negative effects on body and muscle growth, but on the contrary, were beneficial for fish growth during the juvenile phase of this species [41], thus coinciding with other studies on Atlantic cod, *Gadus morhua* [32]. European sea bass, *Dicentrarchus labrax* [42,43], sea bream, and Senegalese sole, *Solea solea* [44,45], were fed with different species of microalgae. The results found by Ayala et al. [41] also showed that *N. gaditana* can be used as an alternative to the excessive use of plant products (mainly soybean) in the diet of gilthead sea bream. 

Different studies have indicated that the influence of the different rearing conditions on muscle growth in early stages of the fish can persist and produce long-term effects on growth mechanisms [46,47,48,49,50,51,52,53]. So far, long-term studies have only been carried out on environmental variables, such as temperature and photoperiod. However, the long-term effect of the diet on muscle growth in fish has not been evaluated yet. Therefore, the present work is aimed to study the possible long-term effect of microalgae-enriched diets supplied in the juvenile phase of seabream specimens. The study has been carried out with specimens from the population studied by Ayala et al. [41]. For this purpose, at the end of the cited experiment [41], the five groups of fish were fed a standard diet (commercial feed) until they reached commercial size (approx. 300 g) and then the analyses of the present work were performed. Thus, the body growth and muscle growth dynamics (hypertrophy/hyperplasia) were analysed at the end of the production cycle of gilthead sea bream specimens.

Muscle cellularity influences the characteristics of the fish fillet, namely textural parameters [34,35,36,37]. Under this perspective, it is worth evaluating the possible long-term influence of microalgae-containing diets on the physiology of muscle growth, and therefore, on the quality of the edible fraction of commercial fish. Currently, our research team is analysing the fillet quality of the specimens of this study to find out the influence of the diet on quality parameters and its possible correlation with muscle parameters.

## 2. Materials and Methods

### 2.1. Rearing Conditions, Experimental Diets, and Sampling

This research was carried out at the Centro Oceanográfico de Murcia-Instituto Español de Oceanografía (COMU-IEO), CSIC from gilthead seabream specimens (*Sparus aurata*) that were obtained from a broodstock breed. These specimens were fed with different experimental diets in the juvenile phase for a three-month period (from ≈ 6.8 to 9.8 months of age). During this previous experiment, the body growth of the juvenile specimens was from ≈ 12 to 50 g (average body weight) and from ≈ 10 to 14 cm (average body length). At the end of the cited experiment, all the animals were transferred to a commercial microalgae-free diet, for 10 months, until the commercial stage of the seabream specimens. At this stage, the specimens were 1.6 years old. In addition, a control group (C group) was fed with a microalgae-free diet. Rearing conditions of the feeding trial during the juvenile stage can be consulted in Ayala et al. [41]. Briefly, the juvenile diets were elaborated with usual aquafeed ingredients, and enriched with the microalgae *Nannochloropsis gaditana* at two inclusion levels (2.5 or 5%)—either raw (R2.5 and R5 groups) or cellulase-hydrolysed (H2.5 and H5 groups). Juvenile specimens were classified in different feeding groups to be fed with the different diets for three months. Table 1 shows the composition of all the diets that were used during the juvenile period of the sea bream population of the present work. Diets were formulated and manufactured at the CEIA_3_-Universidad de Almería facilities (Servicio de Piensos Experimentales, http://www.ual.es/stecnicos_spe (accessed on 23 January 2023)) (Almeria, Spain) by using standard aquafeed extrusion processing procedures. 

The enzymatic hydrolysis was carried out from a sludge containing up to 150 g L^–1^ of biomass. A commercial cellulase (22178, Sigma-Aldrich, Madrid, Spain) was used for 4 h under controlled conditions (pH 5.0 and 50 °C under continuous stirring) providing 2% (w/w) cellulose [41]. Later on, the cellulolytic enzymes were inactivated by heating at 80 °C for 15 min, and then used for manufacturing aquafeeds. As can be seen in Table 1, the percentage of fishmeal is relatively low in all diets (15%), while the percentage of vegetables is very high. In diets R2.5, R5, H2.5, and H5, *N. gaditana* is used as a partial substitute for soybean protein concentrate and wheat meal in order to check whether *N. gaditana* can also be used as an alternative to fishmeal, avoiding excessive use of vegetables in fish diets. However, the percentage of microalgae that was used in the diets was very low (2.5 and 5%) due to the fact that the cost of microalgae is high, and thus we are looking for the lowest dose necessary to obtain an optimal result. In fact, Ayala et al. [41] observed that these low percentages were sufficient to obtain optimal results in sea bream juveniles. The present work investigates the long-term effect of the diet that was used in juvenile specimens. To this end, after the experimental juvenile period, all fish were fed with a commercial diet (Skretting, Burgos, Spain), free of microalgae, for a 10-month additional period. The rearing conditions were like those applied throughout the juvenile stage [41]: 2000-L tanks (1 tank group^–1^) in an open water circuit with natural temperature until reaching 22 °C, at which point the water flow was changed to a closed circuit that enables water cooling, and thus avoids temperature rise during summer. Since then, water was kept around 20 °C. Salinity was 37‰, dissolved oxygen was >5.5 mg L^–1^, and ammonia values were <0.02 mg L^–1^. The photoperiod was natural (ranging from 10 to 13 daylight hours per day) and light intensity ranged from 100 to 150 lx. The number of fish per tank was 75. The groups of the present work were identified with the same nomenclature as the original juvenile batches: C (control, fed with a standard diet, microalgae-free), R2.5 and R5 (fed with raw *N. gaditana* at 2.5 and 5% inclusion level, respectively) and H2.5 and H5 (fed with cellulase pre-hydrolysed *N. gaditana* at 2.5 and 5%, respectively). The R5 group died due to a bacterial infection before sample collection, leaving only four experimental groups for the present study. No mortality was detected in the other experimental groups. 

At the beginning of the experiment, the juvenile specimens were 9.8-month-old, and the mean values of the body weight and body length were 50 g and 14 cm, respectively. After 10 months of receiving the same microalgae-free diet, the specimens reached commercial size (≈27 cm, ≈300 g). Throughout the 10-month experiment, each group was kept in its respective experimental batch, in independent tanks, to study the long-term effects of the juvenile diet on the adult specimens. At the beginning of the experiment, the commercial name of the diet was: D2 Optibream AE 1P. The size of the pellets was 2 mm and the composition was: 48.5% crude protein; 18% crude fat, 6.2% ash, 2.8% cellulose, and 18.5 MJ/Kg digestible energy. Subsequently, the following commercial feed was used: D4 Optibream AE 3P, with a pellet size of 4 mm and the following composition: 44% protein, 20% fat, 6.5% ashes, 3.3% cellulose, and 18.5 MJ/ kg.

The experimental diets were offered *ad libitum* (until apparent satiation) thrice a day: 9:00 a.m., 2:00 p.m., 6:00 p.m. The amount of feed that was ingested by fish was daily recorded in each tank. The feed conversion ratios (total feed being consumed/weight gain) were calculated at the end of the experiment in each tank.

Both at the beginning and at the end of the experiment, all fish from each group (75 fish group^–1^) were collected and sedated with 40 ppm clove oil, and then their body length and body weight were individually recorded. 

### 2.2. Quantitative Analysis of Muscle Growth

Overall, nine specimens from group^−1^ were slaughtered by overdose of anesthesia (60 ppm of clove oil) and then cut transversely to the long body axis. The whole cross muscle section from each fish was photographed and then 5-mm thick whole-body slices were obtained from each fish. Subsequently, these body slices were cut into small blocks and then snap frozen in 2-methylbutane over liquid nitrogen. Later, sections of 8 μm thickness were obtained from those frozen blocks in a cryostat (Leyca CM 1850, Leica Microsistemas SLU, Barcelona, Spain). Subsequently, the sections were stained with haematoxylin-eosin for morphometric muscle studies under light microscope. Muscle growth was quantified by means of a morphometric analysis (Sygma-Scan Pro_5 system, Systat Software Inc., San Jose, CA, USA). The following parameters were measured: the transverse area of the white muscle and the white muscle cellularity (number of white muscles fibers, area and minor axis length of white muscle fibers, and white muscle fibers density). The average size was estimated from ~600 fibers (± 10 sd) located at the intermediate and the apical sectors of the epaxial quadrant of the transversal section of the myotome, according to the methodology described in previous studies in teleosts [37,53,54].

### 2.3. Statistical Analysis

The statistical package SPSS 28 (IBM, New York, NY, USA) was used for the statistical analysis. The data distribution and the homogeneity of variances were analyzed by the Shapiro-Wilk and the Levene’s tests, respectively, for *p* < 0.05. For most of the parameters, both tests showed values of *p* > 0.05 and hence, the analysis of variance (ANOVA) and a post-hoc Tuckey test were used, for *p* < 0.05. However, nonparametric tests (U of Mann-Whitney and Z of Kolmogorov-Smirnov tests) were used in the cases of obtained Shapiro-Wilk test’s values of *p* < 0.05. All the data were expressed as mean ± standard error (SEM).

## 3. Results

### 3.1. Body Parameters and Feed Conversion Ratios

At the beginning of the experiment, the mean values of the body weight and body length of the juvenile specimens were 50 g and 14 cm, respectively. After 10 months of receiving the same microalgae-free diet, the different groups reached the following body growth parameters:

R2.5 versus H2.5 groups: when comparing the body parameters of the commercial-size specimens (1.6-year-old adult specimens) previously fed with raw versus hydrolyzed diets at the same concentration (2.5%), the body weight showed the highest values in the groups previously fed with the hydrolyzed diet (Table 2). However, these differences were not significant (*p* > 0.05) (Table 2).

H2.5 versus H5 groups: when comparing the body parameters from adult specimens previously fed with hydrolyzed diets at different concentrations (2.5 versus 5%), values tended to be higher in the 5% batch, although differences did not reach statistical significance (*p* > 0.05) (Table 2). 

C group: The body parameters were lower in C than in the other groups (*p* > 0.05) (Table 2).

The feed conversion ratios were similar in all the groups: 1.35; 1.33; 1.32, and 1.28 in C, R2.5, R5, H2.5, and H5, respectively. Thus, the values were optimal in all the groups, resulting in an average value of 1.3.

### 3.2. Muscle Parameters

**Figure 1:**
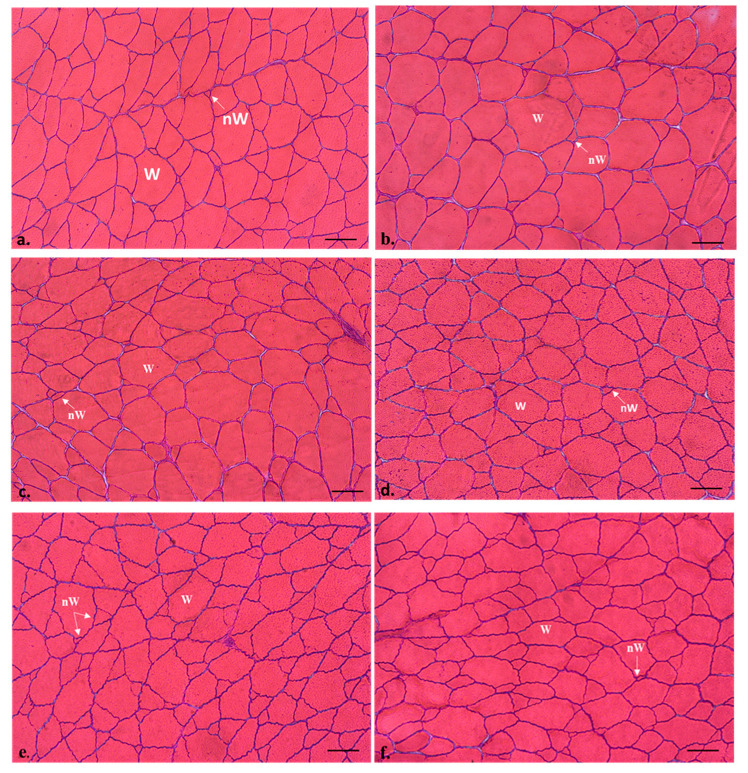
Transverse sections of the white muscle of commercial-size gilthead sea bream (1.6-year-old adult specimens), from C (**a**), R2.5 (**b**,**c**), H2.5 (**d**), and H5 (**e**,**f**) groups. Hematoxylin-Eosin staining. W: white muscle fibres; nW: new white muscle fibres. The white muscle fibres density was higher in the H2.5 and H5 groups (**d**–**f**) than in the other groups (**a**–**c**). Bars 100 µm.

The transverse section of the white muscle of all the specimens displayed the morphological mosaic of fibrillar sizes typical of adult teleosts (Figure 1), with smaller fibers (new generation) interspersed among larger fibers (more mature fibers).

**Figure 2:**
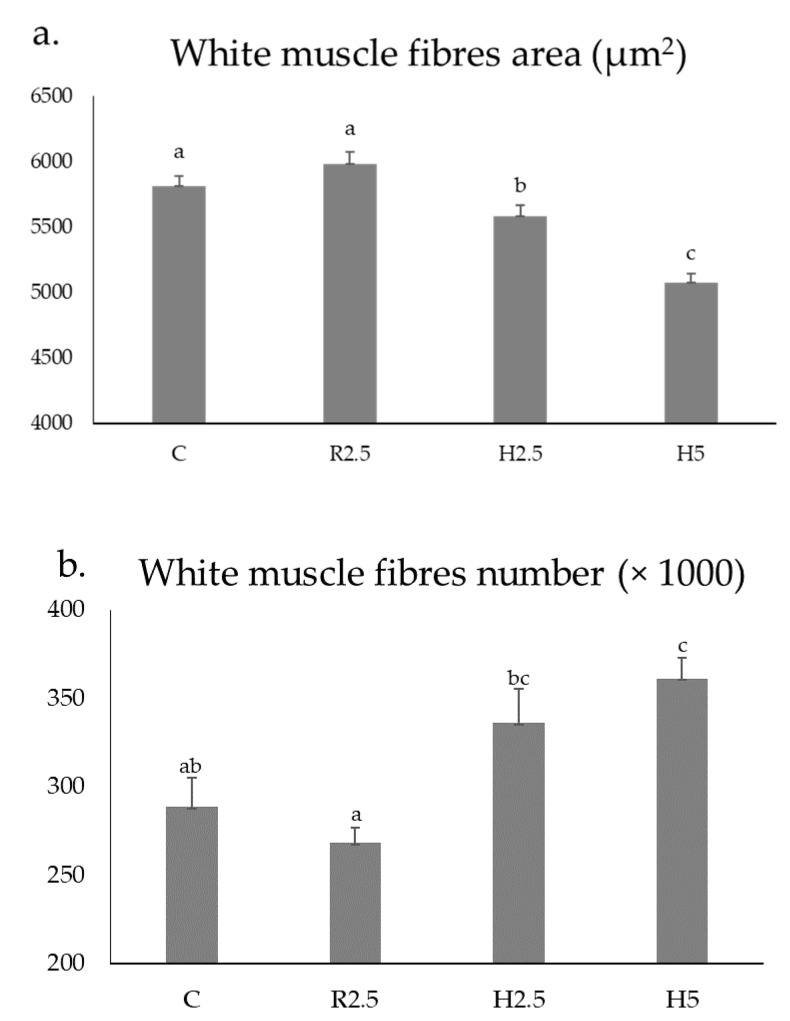
Mean ± SEM values of the area of the white muscle fibres (**a**) and number of the white muscle fibres (**b**) of 1.6-year-old adult specimens of *Sparus aurata* from the C, R2.5, H2.5, and H5 groups. Different lower-case letters indicate significant differences (*p* < 0.05) between groups for each parameter. Mean values were obtained from nine specimens per group.

R2.5 versus H2.5 groups: when comparing the muscle parameters of the adult specimens previously fed with raw versus hydrolyzed diets at the same concentration (2.5%), the hydrolyzed diets increased the values of the transverse area of the white muscle (Table 3). Regarding the muscle cellularity, the raw diets increased the size of the fibres (hypertrophy values: area and minor axis length of the white muscle fibers) (*p* < 0.05) (Table 3, Figure 2a), whereas the hydrolyzed diets increased the hyperplasia values (white muscle fibres number) (*p* < 0.05) (Figure 2b) and the fibrillar density (number of white fibres mm^−2^) (Table 3; Figure 1b–d).

H2.5 versus H5 groups: when comparing the muscle parameters of the adult specimens previously fed with hydrolyzed diets at different concentrations (2.5 versus 5 %), the highest concentration increased the values of the transverse area of the white muscle (Table 3), as well as the hyperplasia and the fibrillar density. In contrast, the lowest concentration of the hydrolyzed diets increased the hypertrophy values of the area and minor axis length of the white muscle fibers (*p* < 0.05) (Table 3; Figure 1d–f; Figure 2).

C group: hypertrophy values of white muscle fibers (area and minor axis length) in C were similar to those found in the R2.5 group and higher than those measured in the H2.5 and H5 groups (Table 3, Figure 1a, Figure 2a). Hyperplasia values of C were similar to those found in R2.5 and H2.5, but significantly lower than those found in H5 (Figure 2b).

## 4. Discussion

### 4.1. Long-Term Influence of the Juvenile Diet with N. gaditana on Body Growth of Adult Specimens of Gilthead Seabream

Fish muscle is plastic in its response to environmental conditions: feeding regime, temperature, photoperiod, salinity, etc. Hence, the external factors influence the number and size of red and white muscle fibres [54,55,56,57,58,59,60]. This influence can persist and cause long-term effects [46,47,48,49,50,51,52,53]. In this context, the present work was carried out to determine the long-term effect of a juvenile feeding regime with raw and hydrolyzed *N. gaditana* in the fish muscle of seabream at commercial stage. 

The results of the present study showed similar and optimal feed conversion ratios in all groups. In relation to the body parameters, the highest body weight values were found in the H5 group, whereas the lowest values of this parameter were observed in the control group. This differs from what was previously found by Ayala et al. [41] in the juvenile phase of this population, in which the highest body weight values were found in R2.5, with no significant influence of enzymatic hydrolysis of the microalgae on fish growth. However, during the juvenile phase of this population, the H5 group showed higher percentage of small and medium white muscle fibers than the other groups [41]. In teleost, the presence of a higher proportion of smaller white fibers implies greater growth potential [39,40,61], which might well explain the trend towards a higher growth rate in the H5 group specimens of the present work. On the other hand, it is necessary to indicate that, while three tanks per group were investigated in the juvenile phase [41], only one tank per group was studied in the present work, which could also influence the results.

### 4.2. Long-Term Influence of the Juvenile Diet with N. gaditana on Muscle Growth Parameters of Adult Specimens of Sparus Aurata

The results indicate a long-term effect of the juvenile diet of gilthead seabream specimens on the subsequent dynamic of muscle growth, reaching the greatest hypertrophy values in control and R2.5 groups, and the highest hyperplasia values in H2.5 and H5 groups at commercial stage (1.6-year-old fish). The long-term effect of different rearing conditions, such as temperature and photoperiod, has been observed in previous studies in teleosts [48,49,50,51]. According to those studies, it seems that both temperature and photoperiod in previous rearing phases exert an “imprinting” effect on the muscle fibres, which determines the dynamics of muscle growth in later stages of the life cycle. This seems to occur through molecular mechanisms acting on the proliferation of myogenic precursor cells [51]. However, so far, no studies on the long-term effect of the diet on the fish muscle growth are known. The present work, therefore, provides evidence for the first time that the diet also has a long-term effect on growth. Given that feed is one of the most determining factors influencing production costs, the fact that small percentages of microalgae in the diets of juvenile fish benefit subsequent growth can help the aquaculture sector to optimize farming and improve profitability.

The different cellularity (hypertrophy/hyperplasia) among the different groups in the present study evidences the plasticity of teleosts muscle under diverse feeding regimes, similarly to what had been observed in teleost species under different rearing conditions [37,53]. In general, hyperplasia is associated with faster growth than hypertrophy [61,62]. Our results agree with this trend in muscle dynamics. Thus, H2.5 and H5 groups showed the highest values of fibrillar hyperplasia and density, which paralleled the higher values of transverse area of white muscle as well as faster growth in both groups than C and R2.5 groups. Furthermore, this effect was dose-dependent, as the higher inclusion levels of hydrolyzed microalgae diet increased the cited parameters. On the contrary, C and R2.5 groups displayed the highest values of fibrillar hypertrophy, together with lower values of white muscle transverse area as well as a slower growth rate than H2.5 and H5 groups. This seems to indicate that the enzymatic hydrolysis of *N. gaditana* could improve the assimilation of microalgae compounds in the diet of juvenile specimens, as has been suggested by other authors [63], which in turn could favour the generation of muscle fibres and their potential growth.

Considering that the different cellularity (hypertrophy/hyperplasia) influences fillet quality—mainly the texture—and the correlation between higher muscle fibre density and fillet firmness [34,35,36,37], we hypothesise that the H5 and H2.5 groups would have higher muscle firmness and improved fillet textural quality than the other groups. The latter will be verified by a complete quality analysis, which is being carried out by our research group.

For aquaculture production, achieving the desired growth rate and ideal final quality is very important in terms of economic sustainability and improved quality of the final product. Although further studies on fillet quality will be necessary, the results of this experiment point to the possibility of optimising the final growth of seabream to commercial size and likely to influencing quality parameters related to muscle structure by using microalgae in juvenile diets, opening the way to a promising option for both fish farmers and the feed manufacturing industry. 

## 5. Conclusions

1. The results indicate a long-term effect of diets for juvenile gilthead seabream on muscle growth dynamics in later stages of culture. Thus, 1.6-year-old adult specimens of gilthead seabream previously fed with H5 and H2.5 diets for a period of 3 months in the juvenile phase showed the highest values of muscle hyperplasia, while adult fish that previously received C and R2.5 diets showed the highest values of muscle hypertrophy. These results were dependent on the dose of hydrolyzed microalgae in juvenile diets, with higher hyperplasia values at higher inclusion levels.
2. The fish growth increased parallel to the generation of muscle fibres in such a way that, at the same age (1.6-year-old specimens), the highest body weight and white muscle transverse area values were reached in the groups that showed the highest muscle hyperplasia values (H5 and H2.5).
3. The highest values of hyperplasia and fibrillar density of the H2.5 and H5 groups seem to indicate a better assimilation of *N. gaditana* when the microalgal biomass was pre-treated with cellulase prior to be included in the diets of the juveniles, which resulted in a higher growth potential. These data suggest a higher fillet firmness, which is currently being analysed by our research group.
4. As a general conclusion, the present work shows that low percentages of *N. gaditana* in the diet of sea bream specimens during a short period of their juvenile phase can improve their long-term growth and, therefore, optimize their culture.

## Figures and Tables

**Table 1:** Ingredient composition of the experimental diets that were used during the juvenile period of sea bream specimens of the present work. Data are expressed in g kg^–1^ dry matter.

Ingredient Composition (g kg^−1^ Dry Matter)	Diets				
	C	R2.5	R5	H2.5	H5
Fish meal LT94 ^1^	150	150	150	150	150
Raw *N. gaditana*		25	50		
Hydrolysed *N. gaditana*				25	50
Squid meal ^2^	20	20	20	20	20
CPSP90 ^3^	10	10	10	10	10
Krill meal ^4^	20	20	20	20	20
Gluten meal ^5^	150	150	150	150	150
Soybean protein concentrate ^6^	400	388	373	388	373
Fish oil ^7^	114	110	105	110	105
Soybean lecithin ^8^	10	10	10	10	10
Wheat meal ^9^	54	45	40	45	40
Choline chloride ^10^	5	5	5	5	5
Betain ^11^	5	5	5	5	5
Lysine ^12^	15	15	15	15	15
Methionine ^13^	6	6	6	6	6
Vitamin and mineral premix ^14^	20	20	20	20	20
Vitamin C ^15^	1	1	1	1	1
Guar gum ^16^	20	20	20	20	20

^1^ 694 g kg^−1^ crude protein, 123 g kg^–1^ crude lipid (Norsildemel, Bergen, Norway); ^2–4^ Bacarel (UK); ^5^ 780 g kg^–1^ crude protein (Lorca Nutricion Animal SA, Murcia, Spain); ^6^ Fish protein hydrolysate, 650 g kg^−1^ crude protein, 80 g kg^−1^ crude lipid (DSM, France); ^7^ AF117DHA (Afamsa, Spain); ^8^ P700IP (Lecico, DE); ^9^ Local provider (Almería, Spain); ^10–13^ Lorca Nutricion Animal SA (Murcia, Spain); ^14^ Lifebioencapsulation SL (Almería, Spain): Mix of vitamins (A, D3, E, K3, B1, B2, B6, B9, B12, H) and minerals (Co, Cu, Fe, I, Mn, Se, Zn, Ca). The proportions of these components can be consulted in Ayala et al. [43]; ^15,^ TECNOVIT, Spain; ^16^ EPSA, Spain.

**Table 2:** Body growth parameters of adult specimens of *Sparus aurata*.

	1.6-Year-Old *Sparus aurata* Specimens			
Groups	C	R2.5	H2.5	H5
BL (cm)	26.92 ^a^ ± 0.16	27.11 ^a^ ± 0.15	26.97 ^a^ ± 0.14	27.1 ^a^ ± 0.12
BW (g)	302.2 ^a^ ± 5.98	309.6 ^a^ ± 5.14	310.6 ^a^ ± 5.11	314.9 ^a^ ± 4.81

Parameters: Body length (BL), body weight (BW). Different lower-case letters superscripts among groups indicate significant differences (*p* < 0.05) for each parameter. Values are expressed as mean ± SEM. Mean values were obtained from 75 fish per group.

**Table 3:** Muscle growth parameters of adult specimens of *Sparus aurata*.

	1.6-Year-old *Sparus aurata* Specimens			
Groups	C	R2.5	H2.5	H5
B (mm^2^)	1656.73 ^a^ ± 65.5	1639.21 ^a^ ± 85.78	1806.87 ^a^ ± 62.50	1819.04 ^a^ ± 69.56
D (μm)	77.61 ^a^ ± 0.59	78.56 ^a^ ± 0.64	76.42 ^b^ ± 0.60	72.60 ^c^ ± 0.55
Dens	174.24 ^ab^ ± 7.16	160.09 ^a^ ± 9.45	183.26 ^ab^ ± 10.87	200.74 ^b^ ± 10.34

Parameters: Transverse area of the white muscle (B); minor axis length of white muscle fibers (D); white muscle fibrillar density (number of white muscle fibers mm^–2^) (Dens). Different lower-case letters superscripts among groups indicate significant differences (*p* < 0.05) for each parameter. Values are expressed as mean ± SEM. Mean values were obtained from nine specimens per group.

## Data Availability

The data presented in this study are available in this article.

## References

[1] Gatlin D.M., Barrows F.T., Brown P., Dabrowski K., Gaylord T.G., Hardy R.W., Herman E., Hu G., Krogdahl Å., Nelson R. (2007). Expanding the utilization of sustainable plant products in aquafeeds: A review. Aquac. Res..

[2] Hardy R.W. (2010). Utilization of plant proteins in fish diets: Effects of global demand and supplies of fishmeal. Aquac. Res..

[3] Turchini G.M., Trushenski J.T., Glencross B.D. (2019). Thoughts for the future of aquaculture nutrition: Realigning perspectives to reflect contemporary issues related to judicious use of marine resources in aquafeeds. N. Am. J. Aquac..

[4] Collins S.A., Øverland M., Skrede A., Drew M.D. (2013). Effect of plant protein sources on growth rate in salmonids: Meta-analysis of dietary inclusion of soybean, pea and canola/rapeseed meals and protein concentrates. Aquaculture.

[5] Yaghoubi M., Mozanzadeh M.T., Marammazi J.G., Safari O., Gisbert E. (2013). Dietary replacement of fish meal by soy products (soybean meal and isolated soy protein) in silvery-black porgy juveniles (*Sparidentex hasta*). Aquaculture.

[6] Shah M.R., Lutzu G.A., Alam A., Sarker P., Chowdhury M.K., Parsaeimehr A., Daroch M. (2018). Microalgae in aquafeeds for a sustainable aquaculture industry. J. Appl. Phycol..

[7] Izquierdo M.S., Montero D., Robaina L., Caballero M.J., Rosenlund G., Ginés R. (2005). Alterations in fillet fatty acid profile and flesh quality in gilthead seabream (*Sparus aurata*) fed vegetable oils for a long term period. Recovery of fatty acid profiles by fish oil feeding. Aquaculture.

[8] Montero D., Grasso V., Izquierdo M.S., Ganga R., Real F., Tort L., Caballero M.J., Acosta F. (2008). Total substitution of fish oil by vegetable oils in gilthead sea bream (*Sparus aurata*) diets: Effects on hepatic Mx expression and some immune parameters. Fish Shellfish Immunol..

[9] Matos E., Dias J., Dinis M.T., Silva T.S. (2007). Sustainability vs. Quality in gilthead seabream (Sparus aurata L.) farming: Are trade-offs inevitable?. Rev. Aquac..

[10] Estruch G., Collado M.C., Monge-Ortiz R., Tomás-Vidal A., Jover-Cerdá M., Peñaranda D.S., Pérez Martínez G., Martínez-Llorens S. (2018). Long-term feeding with high plant protein based diets in gilthead seabream (*Sparus aurata*, L.) leads to changes in the inflammatory and immune related gene expression at intestinal level. BMC Vet. Res..

[11] López C.V., García M.C., Fernández F.G., Bustos C.S., Chisti Y., Sevilla J.M. (2010). Protein measurements of microalgal and cyanobacterial biomass. Bioresour. Technol..

[12] Brown M.R., Jeffrey S.W., Volkman J.K., Dunstan G.A. (1997). Nutritional properties of microalgae for mariculture. Aquaculture.

[13] Chen F., Leng Y., Lu Q., Zhou W. (2021). The application of mi-croalgae biomass and bio-products as aquafeed for aquaculture. Algal Res..

[14] Ju Z.Y., Forster I.P., Dominy W.G. (2009). Effects of supplementing two spe-cies of marine algae or their fractions to a formulated diet on growth, survival and composition of shrimp (*Litopenaeus vannamei*). Aquaculture.

[15] Nayak S., Khozin-Goldberg I., Cohen G., Zilberg D. (2018). Dietary supplementation with ω6 LC-PUFA-rich algae modulates zebrafish immune function and improves resistance to streptococcal infection. Front. Immunol..

[16] Sen Roy S., Pal R. (2015). Microalgae in aquaculture: A review with special references to nutritional value and fish dietetics. Proc. Zool. Soc..

[17] Ferreira M., Ribeiro P.C., Ribeiro L., Barata M., Domingues V.F., Sousa S., Soares C., Marques A., Pousão-Ferreira P., Días J. (2022). Biofortified Diets Containing Algae and Selenised Yeast: Effects on Growth Performance, Nutrient Utilization, and Tissue Composition of Gilthead Seabream (*Sparus aurata*). Front. Physiol..

[18] Becker W. (2003). Microalgae in human and animal nutrition. Handbook of Microalgal Culture.

[19] Kiron V. (2012). Fish immune system and its nutritional modulation for preventive health care. Anim. Feed Sci. Technol..

[20] Sáez M.I., Galafat A., Vizcaíno A.J., Chaves-Pozo E., Ayala M.D., Arizcun M., Alarcón F.J., Suárez M.D., Martínez T.F. (2022). Evaluation of *Nannochloropsis gaditana* raw and hydrolysed biomass at low inclusion level as dietary functional additive for gilthead seabream (*Sparus aurata*) juveniles. Aquaculture.

[21] Kilian O., Benemann C.S.E., Niyogi K.K., Vick B. (2011). High-efficiency homologous recombination in the oil-producing alga *Nannochloropsis* sp.. Proc. Natl. Acad. Sci. USA.

[22] Tibbetts S.M., Mann J., Dumas A. (2017). Apparent digestibility of nutrients, energy, essential amino acids and fatty acids of juvenile Atlantic salmon (*Salmo salar* L.) diets containing whole-cell or cell-ruptured Chlorella vulgaris meals at five dietary inclusion levels. Aquaculture.

[23] Cerón-García M.C., González-López C., Camacho-Rodríguez J., López-Rosales L., García-Camacho F., Molina-Grima E. (2018). Maximizing carotenoid extraction from microalgae used as food additives and determined by liquid chromatography (HPLC). Food Chem..

[24] Heredia H., Pruvost J., Gonçalves O., Drouin L., Marchal L. (2021). Lipid recovery from Nannochloropsis gaditana using the wet pathway: Investigation of the operating parameters of bead milling and centrifugal extraction. Algal Res..

[25] Kavitha S., Gajendranb T., Saranyaa K., Selvakumarc P., Manivasagana V. (2021). Study on consolidated bioprocessing of pre-treated *Nannochloropsis gaditana* biomass into ethanol under optimal strategy. Renew. Energy.

[26] Hjelm J., Svanbäck R., Byström P., Persson L., Wahlström E. (2001). Diet-dependent body morphology and ontogenetic reaction norms in eurasian perch. Oikos.

[27] Rincón D., Velásquez H., Dávila M., Semprun A., Morales E., Hernández J. (2012). Substitution levels of fish meal by *Arthrospira (=Spirulina) maxima* meal in experimental diets for red tilapia fingerlings *(Oreochromis* sp.. ). Rev. Colomb. Cienc. Pecu..

[28] Hussein E., Dabrowski K., El-Saidy D., Lee B.J. (2012). Enhancing the growth of Nile tilapia larvae/juveniles by replacing plant (gluten) protein with algae protein. Aquac. Res..

[29] Kim S., Rahimnejad S., Kim K., Lee K. (2013). Partial replacement of fish meal with *Spirulina pacifica* in *diets* for Parrot fish (*Oplegnathus fasciatus*). Turk. J. Fish. Aquat. Sci..

[30] Teimouri M., Amirkolaie A., Yeganeh S. (2013). The effects of dietary supplement of *Spirulina platensis* on blood carotenoid concentration and fillet colour stability in rainbow trout (*Oncorhynchus mykiss*). Aquaculture.

[31] Pérez-Velázquez M., Gatlin D., González-Félix M., García-Ortega A. (2018). Partial replacement of fishmeal and fish oil by algal meals in diets of red drum *Sciaenops ocellatus*. Aquaculture.

[32] Walker A., Berlinsky D. (2011). Effects of partial replacement of fish meal protein by microalgae on growth, feed intake, and body composition of Atlantic cod. N. Am. J. Aquac..

[33] Knutsen H.R., Johnsen I.H., Keizer S., Sorensen M., Roques J.A.C., Hedén I., Sundell K. (2019). Fish welfare, fast muscle cellularity, fatty acid and body-composition of juvenile spotted wolffish (*Anarhichas minor*) fed a combination of plant proteins and microalgae (*Nannochloropsis oceánica*). Aquaculture.

[34] Hatae K., Yoshimatsu F., Matsumoto J.J. (1984). Discriminative characterization of different texture profiles of various cooked fish muscles. J. Food Sci..

[35] Hatae K., Yoshimatsu F., Matsumoto J.J. (1990). Role of muscle fibres in contributing firmness of cooked fish. J. Food Sci..

[36] Periago M.J., Ayala M.D., López-Albors O., Abdel I., Martínez C., García-Alcázar A., Ros G., Gil F. (2005). Muscle cellularity and flesh quality of wild and farmed sea bass, Dicentrarchus labrax L.. Aquaculture.

[37] Ayala M.D., Arizcun M., García-Alcázar A., Santaella M., Abellán E. (2015). Long-term effects of the larval photoperiod on the subsequent growth of shi drum *Umbrina cirrosa* L. specimens and the fillet texture at commercial size. Turk. J. Fish. Aquat. Sci..

[38] Fauconneau B., Andre S., Chmaitilly J., Le Bail P.Y., Krieg F., Kaushik S.J. (1997). Control of skeletal muscle fibres and adipose cells in the flesh of rainbow trout. J. Fish Biol..

[39] Weatherley A.H., Gill H.S., Rogers S.C. (1980). The relationship between mosaic muscle fibres and size in rainbow trout (*Salmo gairdneri*). J. Fish Biol..

[40] Weatherley A.H., Gill H.S., Lobo A.F. (1988). Recruitment and maximal diameter of axial muscle fibres in teleosts and their relationship to somatic growth and ultimate size. J. Fish Biol..

[41] Ayala M.D., Galián C., Fernández V., Chaves-Pozo E., García de la serrana D., Sáez I.M., Galafat Díaz A., Alarcón F.J., Martínez T.F., Arizcun M. (2020). Influence of Low Dietary Inclusion of the Microalga *Nannochloropsis gaditana* (Lubián 1982) on Performance, Fish Morphology, and Muscle Growth in Juvenile Gilthead Seabream (*Sparus aurata*). Animals.

[42] Tulli F., Chini Zittelli G., Giorgi G., Poli B.M., Tibaldi E., Tredici M.R. (2012). Effect of the inclusion of dried Tetraselmis suecica on growth, feed utilization and fillet composition of European sea bass juveniles fed organic diets. J. Aquat. Food Prod. Technol..

[43] Tibaldi E., ChiniZittelli G., Parisi G., Bruno M., Giorgi G., Tulli F., Venturini S., Tredici M.R., Poli B.M. (2015). Growth performance and quality traits of European sea bass (*Dicentrarchus labrax*) fed diets including increasing levels of freez-dried Isochrysis sp. (T-ISO) biomass as a source protein and n-3 long chain PUFA in partial substitution of fish derivatives. Aquaculture.

[44] Vizcaíno A., López G., Sáez M., Jiménez J., Barros A., Hidalgo L., Camacho-Rodríguez J., Martínez T., Cerón-García M., Alarcón F. (2014). Effects of the microalga Scenedesmus almerienses as fishmeal alternative in diets for gilthead sea bream, *Sparus aurata*, juveniles. Aquaculture.

[45] Vizcaíno A.J., Saéz M.I., López G., Arizcun M., Abellán E., Martínez T.F., Cerón-García M.C., Alarcón F.J. (2016). Tetraselmis suecia and Tisochrysis lutea meal as dietary ingredients for gilthead sea bream (*Sparus aurata* L.) fry. J. Appl. Phycol..

[46] Ayala M.D., López-Albors O., Gil F., García-Alcázar A., Abellán E., Alarcón J.A., Álvarez M.C., Ramírez-Zarzosa G., Moreno F. (2001). Temperature effects on muscle growth in two populations (Atlantic and Mediterranean) of sea bass. Aquaculture.

[47] Johnston I.A., Cole N.J., Abercromby M., Vieira V.L.A. (1998). Embryonic temperature modulates muscle growth characteristics in larval and juvenile herring. J. Exp. Biol..

[48] Johnston I.A., Manthri S., Smart A., Campbell P., Nickell D., Alderson R. (2003). Plasticity of muscle fibre number in seawater stages of Atlantic salmon in response to photoperiod manipulation. J. Exp. Biol..

[49] Johnston I.A., Manthri S., Bickerdike R., Dingwall A., Luijkx R., Campbell P., Nickel D., Alderson R. (2004). Growth performance, muscle structure and flesh quality in out-of-season Atlantic salmon (*Salmo salar*) smolts reared under two different photoperiod regimes. Aquaculture.

[50] Imsland A.K., Foss A., Koedijk R., Folkvord A., Srefansson S.O., Jonassen R.M. (2007). Persistent growth effects of temperature and photoperiod in Atlantic cod *Gadus morhua*. J. Fish Biol..

[51] Steinbacher P., Marschallinger J., Obermayer A., Neuhofer A., Sänger A.M., Stoiber W. (2011). Temperature-dependent modification of muscle precursor cell behaviour is an underlying reason for lasting effects on muscle cellularity and body growth of teleost fish. J. Exp. Biol..

[52] García de la serrana D., Vieira V.L.A., Andree K.B., Darias M., Estévez A., Gisbert E., Johnston I.A. (2012). Development temperature has persistent effects on muscle growth responses in gilthead sea bream. PLoS ONE.

[53] Campos C., Fernandes J.M.O., Conceição L.E.C., Engrola S., Vousa V., Valente L.M.P. (2013). Thermal conditions during larval pelagic phase influence subsequent somatic growth of Senegaleses ole by modulating gene expression and muscle growth dynamics. Aquaculture.

[54] Johnston I.A., Alderson R., Sandham C., Dingwall A., Mitchell D., Selkirk C., Nickell D., Baker R., Robertson B., Whyte D. (2000). Muscle fibre density in relation to the colour and textural of smoked Atlantic salmon (*Salmo salar* L.). Aquaculture.

[55] Johnston I.A. (1999). Muscle development and growth: Potential implications for flesh quality in fish. Aquaculture.

[56] Ayala M.D., López-Albors O., García-Alcázar A., Abellán E., Latorre R., Vázquez J.M., Ramírez-Zarzosa G., Gil F. (2003). Effect of two termal regimes on the muscle growth dynamics of sea bass, *Dicentrarchus labrax* L.. Anat. Histol. Embryol..

[57] López Albors O., Ayala M.D., Gil F., García Alcázar A., Abellán E., Latorre R., Ramírez Zarzosa G., Vázquez J.M. (2003). 2003. Early temperature effects on muscle growth dynamics and histochemical profile of muscle fibre of sea bass Dicentrarchus labrax L., during larval and juvenile stages. Aquaculture.

[58] Johnston I.A., Alderson D., Sandeham C., Mitchell D., Selkirk C., Dingwall A., Nickell D.C., Baker R., Robertson W., Whyte D. (2000). Patterns of muscle growth in early and late maturing populations of Atlantic salmon (*Salmo salar* L.). Aquaculture.

[59] Johnston I.A., Manthri S., Alderson R., Smart A., Campbell P., Nickell D., Robertson B., Paxton C.G.M., Burt M.L. (2003). Freshwater environment affects growth rate and muscle fibre recruitment in seawater stages of Atlantic salmon (*Salmo salar*). J. Exp. Biol..

[60] Campos C., Valente L.M.P., Conceição L.E.C., Engrola S., Fernandes J.M.O. (2013). Temperature affects methylation of the myogenin putative promoter, its expression and muscle cellularity in Senegalese sole larvae. Epigenetics.

[61] Higgins P.J., Thorpe J.E. (1990). Hyperplasia and hypertrophy in the growth of skeletal muscle in juvenile Atlantic salmon *(Salmo salar*, L.). J. Fish Biol..

[62] Veggetti A., Mascarello F., Scapolo P.A., Rowlerson A. (1990). Hyperplastic and hypertrophic growth of lateral muscle in *Dicentrarchus labrax* (L.): An ultrastructural and morphometric study. Anat. Embryol..

[63] Annamalai S.N., Das P., Thaher M.I.A., Abdul Quadir M., Khan S., Mahata C., Al Jabri H. (2021). Nutrients and Energy Digestibility of Microalgal Biomass for Fish Feed Applications. Sustainability.

